# Supplementary material for: Ethics in healthcare: Knowledge, attitude and practices of nurses in the Cape Coast Metropolis of Ghana
Source: PLoS One. 2022 Feb 16;17(2):e0263557. doi: 10.1371/journal.pone.0263557 (PMC8849504; doi:10.1371/journal.pone.0263557)
Supplement: Attachment — Submitted filename: REVD PONE-D-20-10854 Reviewed 30th July 2020.pdf [file pone.0263557.s001.pdf]

PLOS ONE

Ethics in healthcare: Knowledge, attitude and practices of nurses in the Cape Coast Metropolis of Ghana

--Manuscript Draft--

Manuscript Number:	PONE-D-20-10854
Article Type:	Research Article
Full Title:	Ethics in healthcare: Knowledge, attitude and practices of nurses in the Cape Coast Metropolis of Ghana
Short Title:	Ethics in healthcare
Corresponding Author:	Francis Sambah, MPhil<br>University of Cape Coast<br>Cape Coast, Central Region GHANA
Keywords:	
Abstract:	Background<br><br>Nursing is a profession that deals with the most personal and private aspects of people's lives. Nurses need to know the basic ethical aspects of nursing which is integral in nursing practices. The purpose of the study was to describe the ethical knowledge, attitude and practice of nurses in the Cape Coast Metropolis of Ghana.<br><br>Method<br><br>A survey design was used to collect data from 493 nurses in three selected healthcare facilities in the Metropolis. A structured questionnaire was administered to all categories of nurses in the selected facilities. Frequency counts and multiple regression statistics were used to analyze the data.<br><br>Results<br><br>The results show 78% of nurses possessed a high ethical knowledge, 84% had a positive attitude while 98% were ethical conscious in their practices. The results further show that nurses' professional rank [F (1, 259), 2.35, p=.02] and academic qualification [F (1, 259), 2.67, p=.008] were significant predictors of their ethical knowledge and attitude respectively.<br><br>Conclusion<br><br>Inadequate resources, poor set up of working areas and understaffing are the major barriers to nurses' practice with high ethical standards. The Regional Health Directorate, the Ministry of Health and the Managers in charge of the health facilities need to work together to eliminate these barriers as they have the potential to impact negatively on quality healthcare delivery in the Metropolis.
Order of Authors:	Patience Asare
	Edward W. Ansah
	Francis Sambah, MPhil
Additional Information:	
Question	Response
**Financial Disclosure**<br><br>Enter a financial disclosure statement that describes the sources of funding for the work included in this submission. Review the submission guidelines for detailed	The authors received no specific funding for this work.

Powered by Editorial Manager® and ProduXion Manager® from Aries Systems Corporation

requirements. View published research articles from *PLOS ONE* for specific examples.<br><br>This statement is required for submission and **will appear in the published article** if the submission is accepted. Please make sure it is accurate.<br><br>**Unfunded studies**<br>Enter: *The author(s) received no specific funding for this work.*<br><br>**Funded studies**<br>Enter a statement with the following details:<br>• Initials of the authors who received each award<br>• Grant numbers awarded to each author<br>• The full name of each funder<br>• URL of each funder website<br>• Did the sponsors or funders play any role in the study design, data collection and analysis, decision to publish, or preparation of the manuscript?<br>• **NO** - Include this sentence at the end of your statement: *The funders had no role in study design, data collection and analysis, decision to publish, or preparation of the manuscript.*<br>• **YES** - Specify the role(s) played.<br><br>* typeset	
**Competing Interests**<br><br>Use the instructions below to enter a competing interest statement for this submission. On behalf of all authors, disclose any competing interests that could be perceived to bias this work—acknowledging all financial support and any other relevant financial or non-financial competing interests.<br><br>This statement **will appear in the published article** if the submission is accepted. Please make sure it is accurate. View published research articles from *PLOS ONE* for specific examples.	The authors have declared that no competing interests exist.

**NO authors have competing interests**<br><br>Enter: *The authors have declared that no competing interests exist.*<br><br>**Authors with competing interests**<br><br>Enter competing interest details beginning with this statement:<br><br>*I have read the journal's policy and the authors of this manuscript have the following competing interests: [insert competing interests here]*<br><br>* typeset	
**Ethics Statement**<br><br>Enter an ethics statement for this submission. This statement is required if the study involved:<br><br>• Human participants<br>• Human specimens or tissue<br>• Vertebrate animals or cephalopods<br>• Vertebrate embryos or tissues<br>• Field research<br><br>Write "N/A" if the submission does not require an ethics statement.<br><br>General guidance is provided below. Consult the submission guidelines for detailed instructions. **Make sure that all information entered here is included in the Methods section of the manuscript.**	The study protocols were approved by the Institutional Review Board (IRB) of the University of Cape Coast, Ghana (ID: UCCIRB/CES/2015/1), and permission was granted by the heads of administration of the three healthcare facilities and their unit heads.

Powered by Editorial Manager® and ProduXion Manager® from Aries Systems Corporation

**Format for specific study types**<br><br>**Human Subject Research (involving human participants and/or tissue)**<br>• Give the name of the institutional review board or ethics committee that approved the study<br>• Include the approval number and/or a statement indicating approval of this research<br>• Indicate the form of consent obtained (written/oral) or the reason that consent was not obtained (e.g. the data were analyzed anonymously)<br><br>**Animal Research (involving vertebrate animals, embryos or tissues)**<br>• Provide the name of the Institutional Animal Care and Use Committee (IACUC) or other relevant ethics board that reviewed the study protocol, and indicate whether they approved this research or granted a formal waiver of ethical approval<br>• Include an approval number if one was obtained<br>• If the study involved *non-human primates*, add *additional details* about animal welfare and steps taken to ameliorate suffering<br>• If anesthesia, euthanasia, or any kind of animal sacrifice is part of the study, include briefly which substances and/or methods were applied<br><br>**Field Research**<br><br>Include the following details if this study involves the collection of plant, animal, or other materials from a natural setting:<br>• Field permit number<br>• Name of the institution or relevant body that granted permission	
**Data Availability**<br><br>Authors are required to make all data underlying the findings described fully available, without restriction, and from the time of publication. PLOS allows rare exceptions to address legal and ethical concerns. See the PLOS Data Policy and FAQ for detailed information.	Yes - all data are fully available without restriction

Powered by Editorial Manager® and ProduXion Manager® from Aries Systems Corporation

A Data Availability Statement describing where the data can be found is required at submission. Your answers to this question constitute the Data Availability Statement and **will be published in the article**, if accepted.<br><br>**Important:** Stating 'data available on request from the author' is not sufficient. If your data are only available upon request, select 'No' for the first question and explain your exceptional situation in the text box.<br><br>Do the authors confirm that all data underlying the findings described in their manuscript are fully available without restriction?	
**Describe where the data may be found in full sentences. If you are copying our sample text, replace any instances of XXX with the appropriate details.**<br><br>• If the data are **held or will be held in a public repository**, include URLs, accession numbers or DOIs. If this information will only be available after acceptance, indicate this by ticking the box below. For example: *All XXX files are available from the XXX database (accession number(s) XXX, XXX.).*<br>• If the data are all contained **within the manuscript and/or Supporting Information files**, enter the following: *All relevant data are within the manuscript and its Supporting Information files.*<br>• If neither of these applies but you are able to provide **details of access elsewhere**, with or without limitations, please do so. For example:<br><br>*Data cannot be shared publicly because of [XXX]. Data are available from the XXX Institutional Data Access / Ethics Committee (contact via XXX) for researchers who meet the criteria for access to confidential data.*<br><br>*The data underlying the results presented in the study are available from (include the name of the third party*	Data would be made available upon request.

and contact information or URL).<br>• This text is appropriate if the data are owned by a third party and authors do not have permission to share the data.<br><br>* typeset	
Additional data availability information:	

Click here to download Manuscript Ethics paper.docx

Title: Ethics in healthcare: Knowledge, attitude and practices of nurses in the Cape Coast Metropolis of Ghana

Patience Asare[1], Edward W. Ansah[1] & Francis Sambah[1*]

[1]Department of Health, Physical Education and Recreation, University of Cape Coast, Cape Coast, Ghana

*Corresponding author

Email address

PA: patience.asare@stu.ucc.edu.gh

EWA: edward.ansah@ucc.edu.gh

FS: *francis.sambah@stu.ucc.edu.gh (0000-0003-4245-7028)

Abstract

Background: Nursing is a profession that deals with the most personal and private aspects of people's lives. Nurses need to know the basic ethical aspects of nursing which is integral in nursing practices. The purpose of the study was to describe the ethical knowledge, attitude and practice of nurses in the Cape Coast Metropolis of Ghana.

Method: A survey design was used to collect data from 493 nurses in three selected healthcare facilities in the Metropolis. A structured questionnaire was administered to all categories of nurses in the selected facilities. Frequency counts and multiple regression statistics were used to analyze the data.

Results: The results show 78% of nurses possessed a high ethical knowledge, 84% had a positive attitude while 98% were ethical conscious in their practices. The results further show that nurses' professional rank [F (1, 259), 2.35, p=.02] and academic qualification [F (1, 259), 2.67, p=.008] were significant predictors of their ethical knowledge and attitude respectively.

Conclusion: Inadequate resources, poor set up of working areas and understaffing are the major barriers to nurses' practice with high ethical standards. The Regional Health Directorate, the Ministry of Health and the Managers in charge of the health facilities need to work together to eliminate these barriers as they have the potential to impact negatively on quality healthcare delivery in the Metropolis.

Keywords: Healthcare ethics, Knowledge, Attitudes, Practices, Metropolis, Cape Coast, Ghana

Introduction

Ethical issues in healthcare and nursing are becoming more complex couples with medical advances, and the increased dynamics of the healthcare system [1]. Ethics in healthcare is the standards or principles of moral judgment or actions which provide a methodical system in differentiating right from wrong based on certain beliefs [2]. Thus, the first step for nurses in making ethical decisions is to identify and understand the ethical issues surrounding patient care. Ethical nursing practice involves core ethical responsibilities that nurses are expected to uphold, dating back since 1953 by the International Code of Ethics for Nurses (ICN) [3]. Ethical codes in nursing, as in other professions, serve not only to define nursing "profession" but also outline primary duties, responsibilities and obligations towards their clients [4]. Code of ethics for nurses serves as a guide for carrying out nursing responsibilities in a manner consistent with quality care, ethical obligations of the profession, acceptance of the rights of individuals, and to uphold these rights in practice [5]. Moreover, individual dignity needs to be respected, regardless of who is receiving the care, poor, rich, black, white, Christian, Muslim, male or female [6].

The nursing profession requires knowledge of ethics to guide their performance and to impact positively on the lives of their patients [7], but various studies have shown nurses' weaknesses in the knowledge of ethics and its application in practice [8, 9]. Lack of knowledge of the code of ethics is due to the hasty attention given to the code during education, documents that were not readily available, nurses not having the desire to read after work and lack of good nurses' association. In Ghana, the Nursing and Midwife Council (NMC), a statutory body responsible for nursing and midwifery professions, has a code of ethics. It is meant to inform nurses and midwives of the standard required in the exercise of professional accountability and to inform the public, other professionals

and employers of the standard of professional conduct expected of registered nurses and midwives [6].

Many factors influence how nurses act and conduct their duties ethically: knowledge, attitude, and practice. According to Eren [10], unethical behaviors are related to insufficient personnel, poor working conditions, excessive workload, lack of supervision, and in-service training. However, the majority of nurses may not acknowledge that they exhibit unethical behaviors in their practice. Furthermore, others writers [11, 12, 13] believed that age, gender, level of education, professional qualification, years of experience, rank, ward setting, training, marital status, and institution have significant influence on nurses' level of knowledge, attitude and practice of ethics in their professional duties.

The nursing profession aims to give the highest possible standard of care to patients, requiring a high standard of professional behavior [14]. Studies have shown that nurses lack ethical knowledge, portray negative ethical attitudes, and fail to apply their ethical codes in practice [9]. Nurses in Ghana and the Cape Coast Metropolis have been reported in the media to have fallen short of implementing ethical knowledge, demonstrating a good ethical attitude [6], and observing proper ethical practices in their routine patient care [7, 15]. For example, women who attended antenatal clinics complained about how nurses embarrassed them because they could not attend antenatal appointments or afford some of the items required for delivery [16]. These nurses are often described as unfriendly, stubborn, disrespectful, saucy, cruel, callous and inhuman [17, 18]. Despite these growing incidences of unethical practices of nurses in our health facilities in Ghana and the Cape Coast Metropolis, there is a paucity of literature on the extent of such behaviour and factors that might be influencing such practices. Hence, this study aimed to explore knowledge, attitude, and practices of ethics in nursing practice among nurses in the Cape Coast Metropolis.

Methods

This was a cross-sectional survey involving 264 nurses from three healthcare facilities within the Cape Coast Metropolis (i.e. Cape Coast Teaching Hospital [CCTH], Municipal Hospital [MH] and University of Cape Coast Hospital [UCCH]. The majority, 74% (n=194) were females with only 26% (n=70) being males. Sixty seven percent (n=178) of the nurses were between ages 21 and 30, 24% (n=62) 31 and 40 years, and nine percent (n=24) age 41 or above. Academically, 52% (n=138) of the nurses were diploma holders, 27% (n=71) certificate, and only 21% (n=55) hold bachelor degree or higher. Most 65% (n=172) were of the junior rank category whereas 67% (n=177) had a working experience of less than 5 years. We surveyed 52% (n=138) registered general nurses, 21% (n=55) health assistant clinical, 16% (n=41) CHN/RCN/RMN, and 11% (n=30) registered midwives. A majority (67%, n=177) of the participants worked five years or less while the rest, 63% (n=87).

Dependent Measure

We used a 42-items questionnaire, developed by the researchers, to collect data. The instrument was developed from previous empirical literature on ethics and the code of ethics of NMC. The questionnaire solicited information on knowledge, attitude, and practice of ethics, and was two sections, A and B. Section A made up of six multiple choice items, solicited nurses' background characteristics such as sex, age, professional qualification, academic qualification, years of working experience and rank. Section B measured knowledge, attitude, and practice of ethics in nursing practice, using 24 items. This section also collected information on the nurses' sources of knowledge (Q29) and barriers (Q30) to their ethical practice. The section was subcategorized into I, II and III, with I measuring knowledge about ethical practices (Q7-Q16), which the participants responded as yes=3; no=2; do not know =1. Subsection II contained (Q17-Q22) that solicited information about nurses' ethical attitudes, where participants responded strongly disagree =1; disagree =2; agree =3 and strongly agree =4. Furthermore, subsection III (Q23-Q28) measured the extent of ethical practice among nurses, with the

responded categories of never =1, seldom =2, occasionally =3 and always =4. Section B yielded separate scores of knowledge, attitude and practice of ethics by nurses, where high scores or mean reflect maximum performance on that construct.

Copies of the instrument were put in unsealed envelopes and deposited with the ward in-charges who did the onward distribution to the nurses under them. The nurses were given three days to fill the instrument and returned them to their in-charges for retrieval by the researchers. An informed consent form was attached to the instrument, which the participants who agreed to participate signed. The questionnaire was given to five nurses at the Adisadel Health Centre and further to two Senior Research Assistants at the Department of Health, Physical Education and Recreation (HPER) for their evaluation. We further administered the instrument to 79 conveniently sampled nurses from Asankrangwa Catholic Hospital and Samartex Hospital at Samreboi both in the Wasa Amenfi West District in the Western Region of Ghana. This group of nurses did not take part in the main study. The instrument yielded an internal consistency reliability value of 0.73. The study protocols were approved by the Institutional Review Board (IRB) of the University of Cape Coast, Ghana (ID: UCCIRB/CES/2015/1), and permission was granted by the heads of administration of the three healthcare facilities and their unit heads.

Data analysis

Frequency and percentage count were used to determine nurses' level of knowledge, attitude and practice, and barriers to professional ethical conduct at work. Knowledge was measured initially as yes, no and I don't know and later categorized into two putting no and I don't know together for the analysis. Nurses' attitude towards ethical issues at work was also recategorized as positive or negative, from the initial four-point scale. The extent of ethical practice was classified as never, seldom, occasionally and always. Moreover, barriers to the practice of ethics among nurses were analysed and reported with the various items listed on the instrument. In addition, multiple regression

analyses were conducted to provide answers to determine the extent to which background characteristics of nurses influence their knowledge, attitudes, and the extent of ethical practice at their various units of work. Statistical significance levels were determined at a co-efficient alpha of less than .05 [19, 20].

Results

The results show 77.7% (n=205) recorded high knowledge, 66.6% (n=44) moderate whereas 5.7% (n=15) low. Again, majority, 83.7% (n=221) of the nurses showed positive attitude while 16.3% (n=43) reported negative attitude. Furthermore, the results reveal that 98% (n= 258) of the nurses had high practice of ethical standards at work while 2.3% (n=6) did not (see Table 1). The results on barriers to ethical practices reveal that more than half (71%, 63%, and 60%) of the nurses indicated inadequate resources, poor set up of working areas and understaffing (among many others) prevent them from practicing good ethics (see Table 1).

Table 1: Distribution of Nurses' Level of Ethical Knowledge, Attitudes and Practices

	No.	%
Knowledge		
High	205	77.7
Moderate	44	16.6
Low	15	5.7
Attitude		
Positive	221	83.7
Negative	43	16.3
Practice		
Adequate	258	97.7
Inadequate	6	2.3
Barriers		
Inadequate resources	187	70.8
Poor set up of working area	165	62.5
Understaffing	159	60.2
Inadequate education about the code of ethics	136	51.5
Conflicts between nurses and doctors	111	42.0
Working with unethical colleagues	111	42.0
Overcrowding	108	40.9
Lack of space	106	40.2
Working with incompetent colleagues	99	37.5
Conflict with superiors	92	34.8
**Total**	**264**	**100**

Multiple regression analysis was conducted to predict nurses' ethical knowledge with sex, age, rank and academic qualification. The model revealed a significant prediction with the variables, F (4, 259), 17.41, p=.001, accounting for 9% of the variance in nurses' knowledge of ethics. The analysis further indicated that participants' professional rank was the only independent variable significantly predicting nurses' ethical knowledge F (1, 259), 2.35, p=.02, and that nurses' professional rank accounted for 48% of the variations in their knowledge of ethics (see Table 2).

Table 2: Regression of Sex, Age, Academic Qualification and Rank on Nurses' Level of Ethical Knowledge

	B	R	R^2	β	t	p-value
Constant	6.69	.30	.09		17.41	.001
Sex	.00			.00	.02	.985
Age	.00			.01	.16	.874
Academic qualification	.17			.12	1.57	.119
Rank	.48			.21	2.35	.020

df (4, 259); F (17.41)

Multiple regression analysis was conducted to test the extent to which age, sex, rank, professional qualification, academic qualification, and working experience variables predicting nurses' influence their attitude towards the practice of ethics at work. Initial analysis indicated high correlations between age and working experience (r=.91), academic qualification and professional qualification (r=.70). Therefore, age was removed and the regression analysis was based on sex, academic qualification, professional qualification, rank, working experience, and knowledge. The results revealed a significant model predicting nurses' attitude, F (5, 258), 18.02, p=.001, accounting for a 6% variation on nurses' attitudes towards ethics. The analysis further indicated that nurses' academic qualification was the only independent variable significantly predicting nurses' attitude F (1, 259), 2.67, p=.008, accounting for about 27% of the change in their attitude towards ethical practice (see Table 3).

Table 3: Regression of Sex, Academic Qualification, Professional Qualification, Rank and Working Experience on Nurses' Attitude towards Ethical Practice

	B	R	R^2	β	t	p-value

Constant	17.41	.25	.06		18.02	.001
Sex	−.23			−.03	−. 55	.585
Academic qualification	1.12			.27	2.67	.008
Professional qualification	.01			.01	.11	.916
Rank	−.69			−.11	−1.19	.236
Working experience	.03			.06	.79	427

df (5, 258); *F* (18.02)

Multiple regression was further used to determine the extent of ethical practice among nurses based on their age, sex, academic qualification, professional qualification and rank. Initial analysis indicated a high correlation between academic and professional qualification (*r*=.70), which resulted in deleting the academic qualification from the analysis. The regression analysis shows a significant prediction of the model containing age, sex, academic qualification, professional qualification, and rank, *F* (5, 258), 24.23, *p*=.001, accounting for 2% variation in nurses' ethical practice. However, none of these independent variables was independently statistically significant in determining nurses' ethical practice (see Table 4).

Table 4: Regression of Sex, Academic Qualification, Professional Qualification, Rank and Working Experience on the Extent of Ethical Practice of Nurses

	B	*R*	R^2	β	t	p-value
Constant	21.82	.15	.02		24.23	.001
Age	.05			.15	1.80	.073
Sex	−.13			−.02	−.37	.712
Professional qualification	.12			.11	1.22	.226
Rank	−.25			−.05	−.53	596

df (5, 258); *F*(24.23)

Discussion

The focus of this study was to describe the knowledge, attitude, and ethical practices among nurses in the Cape Coast Metropolis of Ghana. We observed a high level of ethical knowledge among the nurse participants. First, we are of the view that the nurses in this study recognize ethical problems in the course of their practice because of seminars and workshops they attended during the period of practice, as has been reported in other studies [7, 21]. Further, ethics is emphasized across all levels of nursing education

globally and Ghana [6, 22]. Besides, every professional group has established a code of ethics and standards, normally drawn from the ICN, so, it does not matter where the nurse is trained, they are expected to adhere to those ethics [23], which agrees with previous studies in Turkey [10], Spain [21], Australia [24], Italy [25], Iran [26], and Hong Kong [27]. Conversely, other previous findings from Northern India [8], Ethiopia [13], Germany [28], and Nigeria [29] revealed a low to moderate level of ethical knowledge. The reason for these dissimilarities could be attributed to methodological limitations. For example, while the earlier studies [8, 13] used nurses and other healthcare workers at primary facility level, and [28] from national population survey, our participants are nurses from secondary and tertiary facilities. Ethical knowledge is required in the nursing profession to guide nurses in the delivery of health care. Having sufficient ethical knowledge may positively influence the professional conduct of nurses which has been a great public concern in Ghana in recent times [17, 18].

The finding again indicates a high positive attitude towards ethical practice. We reason here that participants are applying their ethical knowledge to practice, which may be influencing their attitude. Moreover, practice experience of these nurses might bring them in contact with various ethical issues resulting in many having positive attitude, as evidenced from other studies in Oceania [11], Africa [12, 13], Asia [27], and Europe [30, 31] which found nurses to have positive attitudes towards ethical practice. Health care delivery in the Cape Coast Metropolis of Ghana may be improved through this positive attitude of nurses which may enhance the recovery rate of patients since negative attitudes compromise patient's attitude and recovery. Research by Norbergh et al. [31] indicated that a positive attitude is important for the wellbeing and quality of care given to patients because such attitude enhances patients' satisfaction and promotes their health and psychological wellbeing.

We further observed adequate ethical practice among nurse participants. The reason may be because participants well understood the issues in ethics that they were

taught during their training and applied the knowledge to professional practice. Moreover, these nurses may have encountered ethical issues and might be familiar with these issues which could inform their adequate practice. Semblance to this finding, other studies conducted in Africa [12, 13] among nurse participants, found their ethical practices to be adequate or good. Ethical practice is essential not only to nursing but to patients care as it underpins the fiber of the practice itself [1, 4]. Moreover, practices deemed unethical can compromise the health of the client, and land the practitioner into a lawsuit, resulting in the revocation of license and/or imprisonment. In contrast, studies from Nigeria [32] and West Indies [33] found inadequate ethical practice among participants, which may be because of the vast period between these studies and our own. For example, the study from Nigeria [32] is 10 years earlier to the current study, which is enough in an evolving industry as health care for some ethical transformations and improvement to take place.

There is an indication also that inadequate resources, poor set up of the working area and understaffing are major barriers to ethical practices among the nurses. Plausibly, most nurses do not get the necessary or basic resources (i.e. consumables, conducive working environment to ensure confidentiality and privacy of clients) and this disincentivized adherence to ethical practices in nursing care. In low-middle-income countries such as Ghana, health care delivery is bedeviled with several barriers, including space, understaffing and provision of resources, to enhance caregiving to patients [18, 34]. For example, evidence [34, 35] indicates that understaffing which may force nurses to abandon opportunities to provide good nursing care to their patients, may also increase workload, absenteeism, turnover, work stress, burnout, and job dissatisfaction [18, 32, 34]. In this case, quality health care delivery may fester unprofessional conduct and could be counterproductive to the health outcomes of patients.

We observed that participants' professional ranks influence their level of ethical knowledge. It is likely that because a majority of the study participants were found within

the junior nurse rank category (i.e. most of them in their youthful years), they may have fresh repertoire of their ethics education from school [21]. It is also sound to theorized that as participants rise from one rank to another, they encounter different ethical dilemmas which may increase their knowledge of ethics, evidence from Nigeria [12], New South Wales [36], and Kenya [37]. Besides, academic qualification positively determined nurses' attitude towards ethical practice at work. Deductively, the majority of the study participants are diplomates, also with a high level of knowledge and a positive attitude. This concurred with a study from Ethiopia [13] that the level of education/academic qualification significantly influence nurses' attitude. Contrarily, other studies in Nigeria [12], Hong Kong [27], and Ireland [31] reported that level of education did not have any significant influence on attitude to practicing ethics at work. Though our study did not draw cause and effect linkage, it is sound to infer that, higher educational qualification has a propensity to positively transformed attitudes.

Conclusions

This study aimed to describe the knowledge, attitude, and practice of ethics among nurses in the Cape Coast Metropolis of Ghana. Though these nurses record high ethical knowledge, possess a positive attitude, and are ethically conscious in their practices, they are faced with inadequate resources, poor set up of the working area and understaffing, limit the extent of treating their clients with high ethical standards. Further, professional rank and academic qualification are key to changing the ethical knowledge and attitude these nurses. Thus, a high level of ethical practice becomes a concern in nursing clients, especially in a relatively weak healthcare system like that observed in Ghana. This is as a result of a cross-section study, and that we do intend to infer cause and effect, generalize the findings and conclusions to the whole Ghana and other healthcare professionals. However, this study further opens the discourse on nurses' ethical knowledge, attitudes, and practices during the healthcare delivery process, in the Cape Coast Metropolis of Ghana.

Recommendations

Nurses need to continually update their knowledge through seminars/workshops to be abreast with ethical issues that arise daily during practice, which could also impact their attitude positively. Nurse managers need to do more of the periodic monitoring of nurses' attitudes to maintain the high image of the profession. The teaching of ethics during the training of nurses needs to be intensified since most of them obtained their knowledge of nursing ethics in the schools before getting to the job. There is the need to address those identified barriers, i.e. provide the needed resources, increase the staff strength, get better working space, to help nurses give the quality of care needed by their patients. Extending this study nationwide will be ideal to get wider views and understanding of the issues studied.

Author Contributions: PA conceived the study. PA and EWA analysed the data. FS, EWA and PA prepared the first draft of the manuscript. All authors proofread the manuscript for intellectual content and approved for publication.

Competing interests: The authors have declared that no competing interests exist

Funding: No funding was received for this study.

References

1. Park M, Jeon SH, Hong HJ, Cho SH. A comparison of ethical issues in nursing practice across nursing units. Nursing ethics. 2014, 21(5), 594-607.
2. Antipuesto DJ. Nursing Ethics. 2010. Retrieved from: http://nursingcrib.com/nursing-notes-reviewer/fundamentalsonursing
3. International Council of Nurses. Code of Ethics for nurses. 2012. Retrieved from: http:/www.icn.ch/images/stories/documents/about/icncode_english
4. Zahedi F, Sanjari M, Aala M, Peymani M, Aramesh K, Parsapour A, Maddah SB, Cheraghi MA, Mirzabeigi GH, Larijani B, Dastgerdi MV. The code of ethics for nurses. Iranian journal of public health. 2013, 42(Supple1);1.

5. Nursing and Midwifery Board of Ireland. Code of professional conduct and ethics for registered nurses and registered midwives. 2013. Draft for consultation purposes. Retrieved from: www.nursingboard.ie/getAttachment.aspx?id=48c95d7f-83e6-4858
6. Nurses and Midwives' Council of Ghana. Curriculum for the semester course unit system of health assistant (clinical) programme. 2006b.
7. Ghana News Agency. Nursing and Midwifery Council holds workshop for nurses in Brong Ahafo. (19, April, 2014).Retrieved from: http://www.ghananewsagency.org/health/nmc-holds-workshop-for nurses-in-brong-ahafo-73849
8. Bhardwaj A, Chopra M, Mithra P, Singh A, Siddiqui A, Rajesh DR. Current Status of Knowledge, Attitudes and Practices towards Healthcare Ethics Among Doctors and Nurses from Northern India-A Multicentre Study. Pravara Medical Review. 2014, 6(2).
9. Tadd W, Clarke A, Lloyd L, Leino-Kilpi H, Strandell C, Lemonidou C, Petsios K, Sala R, Barazzetti G, Radaelli S, Zalewski Z. The value of nurses' codes: European nurses' views. Nursing Ethics. 2006, 13(4);376-93.
10. Eren N. Nurses' attitudes toward ethical issues in psychiatric inpatient settings. Nursing ethics. 2014, 21(3), 359-73.
11. Crothers CE, Dorrian J. Determinants of nurses' attitudes toward the care of patients with alcohol problems. ISRN nursing. 2011.
12. Aliyu D, Adeleke IT, Omoniyi SO, Samaila BA, Adamu A, Abubakar AY. Knowledge, attitude and practice of nursing ethics and law among nurses at Federal Medical Centre, Bida. American Journal of Health Research. 2015, 3(1-1), 32-37.
13. Kassa H, Murugan R, Zewdu F, Hailu M, Woldeyohannes D. Assessment of knowledge, attitude and practice and associated factors towards palliative care among nurses working in selected hospitals, Addis Ababa, Ethiopia. BMC palliative care. 2014, 13(1):6.

14. The Nursing Board. The code of professional conduct for each nurse and midwife. 2000. Retrieved from: www.nursingboard.ie/GetAttachment.aspx?id=8a9adc68-4571-4d44
15. Peacefmonlinenews. Nurses and midwives deliberate on ethical/legal implications of their attitudes. (2014, May 11). Retrieved from: http://news.peacefmonline.com/pages/health/201405/199173.php
16. Adei D, Fiscian YB, Ephraim L, Diko SK. Access to maternal health care services in the Cape Coast Metropolitan Area, Ghana. Current Research Journal of Social Sciences. 2012, 4(1), 12-20.
17. Adofo R., A case of deplorable attitude of Ghana nurses…true story. (2010, October 12). Retrieved from: http://www.ghana web.com/GhanaHomePage/NewsArchive/artikel.ph?ID=195002
18. Korsah KA. Nurses' stories about their interactions with patients at the Holy Family Hospital, Techiman, Ghana. Open Journal of Nursing. 2011, 1(01);1.
19. Ofori R, & Dampson D G. Research methods and statistics using SPSS. 2011. Amakom-Kumasi: Payless Publication Limited.
20. Huck SW. Reading statistics and research (5th ed.). 2008. United State of America: Pearson Education, Inc.
21. Iglesias ME, de Bengoa Vallejo RB. Nurse attitudes in relation to health care ethics and legal regulations for nursing. Acta Bioethica. 2014, 20(2).
22. Cherie A, Mekonen AH, Shimelse T. Introduction to professional nursing and ethics. Addis Ababa University, In collaboration with the Ethiopia Public Health Training Initiative, The Carter Center, the Ethiopia Ministry of Health, and the Ethiopia Ministry of Education, Addis Ababa. 2005.
23. Kozier B, Erb G, Berman A, & Synder S. Fundamentals of nursing: Concepts, process and practice (7th ed.). 2004. USA, Pearson Education, Inc.

24. Jonhstone MJ, Da Costa C, Turale S. Registered and enrolled nurses' experiences of ethical issues in nursing practice. Australian Journal of Advanced Nursing, The. 2004, 22(1), 24.
25. Leuter C, Petrucci C, Mattei A, Tabassi G, Lancia L. Ethical difficulties in nursing, educational needs and attitudes about using ethics resources. Nursing ethics. 2013, 20(3), 348-58.
26. Sheikhtaheri A, Jabali MS, Dehaghi ZH. Nurses' knowledge and performance of the patients' bill of rights. Nursing ethics. 2016, 23(8), 866-76.
27. Yeung MC. Nurses' knowledge, attitudes and roles regarding advance directives in Hong Kong. 2006, 1, 1-0.
28. Eilts-Köchling K, Heinze C, Schattner P, Voß M, Dassen T. Knowledge of professional codes of ethics among nursing professionals. Pflege. 2000, 13(1), 42-6.
29. Oyetunde MO, Ofi BA. Nurses' knowledge of legal aspects of nursing practice in Ibadan, Nigeria. Journal of Nursing Education and Practice. 2013, 3(9):75.
30. Clancy M. Irish Emergency Nurses' Attitudes towards Role Expansion in, and Barriers to, Nurse Prescribing.
31. Norbergh KG, Helin Y, Dahl A, Hellzén O, Asplund K. Nurses' attitudes towards people with dementia: The semantic differential technique. Nursing Ethics. 2006, 13(3), 264-74.
32. Reis C, Heisler M, Amowitz LL, Moreland RS, Mafeni JO, Anyamele C, Iacopino V. Discriminatory attitudes and practices by health workers toward patients with HIV/AIDS in Nigeria. PLoS medicine. 2005, 2(8), e246.
33. Hariharan S, Jonnalagadda R, Walrond E, Moseley H. Knowledge, attitudes and practice of healthcare ethics and law among doctors and nurses in Barbados. BMC Medical ethics. 2006, 7(1):7.
34. Maluwa VM, Andre J, Ndebele P, Chilemba E. Moral distress in nursing practice in Malawi. Nursing ethics. 2012, 19(2), 196-207.

35. Ulrich CM, Taylor C, Soeken K, O'Donnell P, Farrar A, Danis M, Grady C. Everyday ethics: ethical issues and stress in nursing practice. Journal of advanced nursing. 2010, 66(11), 2510-9.

36. van de Mortel TF. Registered and enrolled nurses' knowledge of hepatitis C and attitudes towards patients with hepatitis C. Contemporary nurse. 2004, 16(1-2), 133-44.

37. Ndetei DM, Khasakhala LI, Mutiso V, Mbwayo AW. Knowledge, attitude and practice (KAP) of mental illness among staff in general medical facilities in Kenya: practice and policy implications. African journal of psychiatry. 2011, 14(3), 225-35.
