# Supplementary material for: Ethics in healthcare: Knowledge, attitude and practices of nurses in the Cape Coast Metropolis of Ghana
Source: PLoS One. 2022 Feb 16;17(2):e0263557. doi: 10.1371/journal.pone.0263557 (PMC8849504; doi:10.1371/journal.pone.0263557)
Supplement: Attachment — Submitted filename: Review comments and our Responses.docx [file pone.0263557.s002.docx]

Dear Editor and Reviewers,

On behalf of all authors, I convey our gratitude to you for the critical and constructive review that has led to the improvement of our paper entitled “Ethics in healthcare: Knowledge, attitude and practices of nurses in the Cape Coast Metropolis of Ghana”. We have revised the manuscript based on the comments raised by both the Editor and the Reviewers. We believe the manuscript has improved substantively and will be published in your reputable journal, PlosOne. All the changes have been marked yellow colour in the revised manuscript. Please address all correspondence to me via email at: [**francis.sambah@stu.ucc.edu.gh**](mailto:francis.sambah@stu.ucc.edu.gh)

Thank you

**Editors Comments**

1. **Comment:**

Please ensure that your manuscript meets PLOS ONE's style requirements, including those for file naming. The PLOS ONE style templates can be found at https://journals.plos.org/plosone/s/file?id=wjVg/PLOSOne_formatting_sample_main_body.pdf and <https://journals.plos.org/plosone/s/file?id=ba62/PLOSOne_formatting_sample_title_authors_affiliations.pdf>

**Response:** We have made the necessary corrections in conformity with the PLOS ONE’s style.

2. **Comment:**

In your Methods section, please provide additional information about the participant recruitment method and the demographic details of your participants. Please ensure you have provided sufficient details to replicate the analyses such as: a) the recruitment date range (month and year), b) a description of any inclusion/exclusion criteria that were applied to participant recruitment, c) a table of relevant demographic details, d) a statement as to whether your sample can be considered representative of a larger population, e) a description of how participants were recruited, and f) descriptions of where participants were recruited and where the research took place.

**Response:** We have made efforts to review the method section and included the dates for the study, inclusion and exclusion criteria. In addition, we put the demographics into a table (Table 1).

3. **Comment:**

Please include additional information regarding the survey or questionnaire used in the study and ensure that you have provided sufficient details that others could replicate the analyses. For instance, if you developed a questionnaire as part of this study and it is not under a copyright more restrictive than CC-BY, please include a copy, in both the original language and English, as Supporting Information.

**Response:** We reworked on these, describing the questionnaire and how data was collected.

4. **Comment**:

We suggest you thoroughly copyedit your manuscript for language usage, spelling, and grammar. If you do not know anyone who can help you do this, you may wish to consider employing a professional scientific editing service.

**Response:** We have made the necessary efforts to edit the manuscript and also sought a help from a professional scientific editor.

Comment:

Whilst you may use any professional scientific editing service of your choice, PLOS has partnered with both American Journal Experts (AJE) and Editage to provide discounted services to PLOS authors. Both organizations have experience helping authors meet PLOS guidelines and can provide language editing, translation, manuscript formatting, and figure formatting to ensure your manuscript meets our submission guidelines. To take advantage of our partnership with AJE, visit the AJE website (http://learn.aje.com/plos/) for a 15% discount off AJE services. To take advantage of our partnership with Editage, visit the Editage website (www.editage.com) and enter referral code PLOSEDIT for a 15% discount off Editage services. If the PLOS editorial team finds any language issues in text that either AJE or Editage has edited, the service provider will re-edit the text for free.

Upon resubmission, please provide the following: The name of the colleague or the details of the professional service that edited your manuscript. A copy of your manuscript showing your changes by either highlighting them or using track changes (uploaded as a *supporting information* file). A clean copy of the edited manuscript (uploaded as the new *manuscript* file).

**Response:** We have employed Dr. Jacob Owusu Sarfo and Mr. Frank Mensah for copy edition the manuscript.

**5. Comment:**

We note that you have indicated that data from this study are available upon request. PLOS only allows data to be available upon request if there are legal or ethical restrictions on sharing data publicly. For more information on unacceptable data access restrictions, please see http://journals.plos.org/plosone/s/data-availability#loc-unacceptable-data-access-restrictions.

**Response:** The dataset supporting this manuscript has been uploaded into the Open Science Framework platform DOI 10.17605/OSF.IO/SXMGU

**Comment:**

In your revised cover letter, please address the following prompts:

a) If there are ethical or legal restrictions on sharing a de-identified data set, please explain them in detail (e.g., data contain potentially sensitive information, data are owned by a third-party organization, etc.) and who has imposed them (e.g., an ethics committee). Please also provide contact information for a data access committee, ethics committee, or other institutional body to which data requests may be sent.

**Response:** The data is freely available at Open Science Framework platform via DOI 10.17605/OSF.IO/SXMGU

b) If there are no restrictions, please upload the minimal anonymized data set necessary to replicate your study findings as either Supporting Information files or to a stable, public repository and provide us with the relevant URLs, DOIs, or accession numbers. For a list of acceptable repositories, please see <http://journals.plos.org/plosone/s/data-availability#loc-recommended-repositories>.

**Response:** The dataset supporting this manuscript has been uploaded into the Open Science Framework platform DOI 10.17605/OSF.IO/SXMGU

We will update your Data Availability statement on your behalf to reflect the information you provide.

**6. Comment:**

PLOS requires an ORCID iD for the corresponding author in Editorial Manager on papers submitted after December 6th, 2016. Please ensure that you have an ORCID iD and that it is validated in Editorial Manager. To do this, go to ‘Update my Information’ (in the upper left-hand corner of the main menu), and click on the Fetch/Validate link next to the ORCID field. This will take you to the ORCID site and allow you to create a new iD or authenticate a pre-existing iD in Editorial Manager. Please see the following video for instructions on linking an ORCID iD to your Editorial Manager account: <https://www.youtube.com/watch?v=_xcclfuvtxQ>

**Response:**  The corresponding author and one of the co-authors have added their ORCID iDs.

**Reviewer #1:**

Summary of Review and overall impression

**Comment:**

Ethical issues in nursing is an important area on nursing given that Nurses interact with patients at very delicate point in their lives when they are vulnerable to abuse of their rights. However, there are important issues that need to be included. E.g the specific ethical principles, theories etc. that are of importance to nursing. The authors need to demonstrate how these principles are integrated in the International Council of Nurses guidelines and operationalized in National nurses’ regulatory body and curricular for nurses in Ghana.

**Response:**  We have attempted to address the issues raised here.

**Comment:**

There is also need to describe how the ethical guidelines for nurses relate to the specific areas of Ghana constitution.

**Response:**  We made changes to the literature reflecting these issues.

**Comment:**

My general view is that assessment of knowledge, attitude and practice of nurses in the area of ethics is an important issue but the manuscript needs to be revised substantially before publication as follows:

Introduction: There is need for major revision to give description in the subject matter of ethics, magnitude of the problem, the negative effects and gaps in Knowledge, attitudes and practice that justify the study.

**Response:**  We have made changes to improve the introductory part of the manuscript.

**Comment:**

The literature review does not demonstrate logical arguments that justify the study

**Response:** We have made changes to improve the literature.

**Comment:**

Methodology: There are major methodological issues of concern indicated on the specific areas (see comments in the manuscripts for detailed suggestions).

**Response:**  We have made substantive revision to the method section to reflect the comments made by the reviewers.

**Comment:**

Results and Discussion: Need to be revised as indicated in the comments within the document.

Generally-there is need to revise the manuscript for logical flow.

**Response:** We have revised the results and discussion for logical flow in the manuscript.

**Comment:**

Declaration of competing interest-I declare that I do not have any conflicting interest

**Response:** We added that to the manuscript.

**Reviewer #2:**

**Comment:**

This is an interesting and informative paper on a geographically understudied area of nursing practice. As I am a qualitative researcher, I cannot comment on the regression analysis specifically. Outside of that, the study design and methodology seem sound and the results support the conclusions, with one caveat:

**Response:** We appreciate your comments.

**Comment:**

I would encourage the authors to consider and discuss the difference between 1) 'ethics' in a general social sense, 2) professional ethics as specific to nursing, and 3) the particular written national codes of ethics they discuss in the paper. They should make clear that when discussing 'nurse's knowledge of ethics', they mean the third sense, namely formal written codes of conduct. I'm saying this because it could be well possible that rather than having 'no ethics at all', as the authors seem to imply, the nurses in question simply operate on a different (maybe not formal/bureaucratic) understanding of ethics, which then conflicts with professional standards. For example, their treatment of women in antenatal clinics may not be in tune with formal ethical standards at the hospital - but is it possible that it conforms to a 'local' ethics which demands that women attend the clinic and pay for the items needed there? These distinctions might make for an interesting follow-up study, but as it stands, the data does not support the claim that nurses do not have any knowledge of any ethics at all - just that they do not have knowledge of the relevant formal codes applying to their jobs.

**Response:**  We appreciate the comments. We made changes to reflect ethical practices of nurses in the Ghanaian context.

**Comment:**

I would also recommend to have the article professionally edited if at all possible, to eliminate potential misunderstandings due to language use (e.g. p 57 ln 59 - 'couples'?).

**Response:** We deleted the word “couples” to make the sentence readable.

**Comment:**

The authors state that the original data will be made available upon request, but it appears that PLOS ONE requires data to be deposited in a public depository for the paper to be accepted. Once these points are addressed, I warmly recommend the paper for publication.

**Response:** We are making the necessary efforts to upload the data.

**Reviewer #3:**

**Comment:**

1. Page Number - PN (3) Line Number - LN (58): - Is this issue of unethical practice among nurses a global one or just specific to some places? Or are there other forms in other places?

**Response:** We have attempted to rewrite the sentence for clarity.

**Comment:**

2. PN (3) LN (75-79): - Where were these studies conducted? Is this a global problem or localized? You need to be specific here. There might be a need to present existing global studies especially from the western world in relation to the unethical nursing practices. LN 79 - Please change to ‘Midwifery’.

**Response:** We have rewritten the sentences to make better understanding of the issues raised.

**Comment:**

3. PN (4), LN (85-86): - This sentence is not clear. How does the attitude and the practice of nurses influence how they act and conduct their duties? Or that the nurses’ knowledge of the ethics of their profession influenced their attitudes and practices? LN (95-97): - Is this referring to the nurses in Ghana?

**Response:**  We have improved the literature.

**Comment:**

4. PN (5), LN (116): - Change to ‘bachelor’s degree’

**Response:** We made the change accordingly.

**Comment:**

5. PN (6), LN (144-147): - This appears confusing. Can you please elaborate on this sentence especially regarding the evaluation by the two senior research assistants? Was this part of the analysis? LN (149-150): This is not clear. Which of the group of nurses did not take part in the main study? Why were they not involved in the main study? Was there a pilot test?

**Response:** We have rewritten this part for clarity. This was a pilot test of the questionnaire.

**Comment:**

6. PN (10), LN (273): - Change to ‘…and in Ghana’ LN (294): Please rewrite this.

**Response:** We made the change accordingly.

**Comment:**

7. PN (11), LN (305-307): - Yes, this is true. However, do the nurses in Ghana or any of the African countries face lawsuits for unethical practices? Are the people aware of their rights regarding the unethical practices of the nurses who take care of them? Have you also considered the issues of quackery in the nursing profession, whereby people assumed that everyone working in health facilities wearing ‘white uniforms’ are nurses? In essence, what role does quackery play in the unethical practices observed among the nurses in Ghana?

**Response:** We have made the necessary improvement to this part of the discission.

**Comment:**

LN (314 - 320): From your finding, and with the high percentage of ethical practices among nurses in Ghana, what could be the explanation for these inhibiting factors raised here and the high percentage of ethical practices among the nurses interviewed in this study?

**Response:** We have made modifications to this.

**Comment:**

8. PN (12), LN (332-333): - You need to expatiate more on this issue. Are diplomate nurses found to be more involved in unethical practices as a result of their low level of education? Or what?

From this study findings, nurses who have diploma in nursing also have high level of knowledge about the ethics of nursing profession. This appears to contradict earlier studies in relation to this.

**Response:** We have expatiated this appropriately.

**Comment:**

LN (349-350): How can the findings from this study be generalized to other health care professionals? Are they also influenced by the same factors? Are there studies that suggest that they also practice unethically? Which of the health care professionals do you want to generalize your findings to?

**Response:** We have restated this section appropriately to reflect that we refereeing to only nurses.

**Comment:**

LN (350-353): Further study should be encouraged to identify specific nursing specialties where there are reports of unethical practices. There is also a need to identify the specific aspects of the nursing ethics where unethical practices have been identified among nurses. Studies should be done to identify if there are complaints about unethical practices among other health care professionals.

**Response:** We have modified this appropriately.

**Comment:**

9. PN (13), LN (357-358): There should be sanctions and penalties for nurses who are reported and confirmed to be involved in unethical practices.

**Response:** We added a sentence that calls for sanctions and penalties for nurses with unethical practices.
