# Supplementary material for: Ethics in healthcare: Knowledge, attitude and practices of nurses in the Cape Coast Metropolis of Ghana
Source: PLoS One. 2022 Feb 16;17(2):e0263557. doi: 10.1371/journal.pone.0263557 (PMC8849504; doi:10.1371/journal.pone.0263557)
Supplement: Attachment — Submitted filename: Ethics paper_Review Comments_14_9_2021.docx [file pone.0263557.s003.docx]

Department of Health, Physical Education and Recreation

University of Cape Coast

14^th^ September 2021

Dear Editor and Reviewers,

On behalf of all authors, I convey our gratitude to you for the critical and constructive review that has led to the improvement of our paper entitled **“Ethics in healthcare: Knowledge, attitude and practices of nurses in the Cape Coast Metropolis of Ghana”.** We have revised the manuscript based on the comments raised by both the Editor and the Reviewers. We believe the manuscript has improved substantively and will be published in your reputable journal, PlosOne. All the changes have been marked with red colour in the revised manuscript. Please address all correspondence to me via email at: [sambahfrancis80@gmail.com](mailto:sambahfrancis80@gmail.com)

Thank you

Yours sincerely

Francis Sambah

(Corresponding author)

**Response to Editor and Reviewers comments**

“line 34: Method – survey design is not appropriate term. it is preferable to use cross-sectional design:

Response: We have made the correction as appropriate (line 34).

• “line 38: high ethical knowledge is not appropriate – it is better to state good knowledge”

Response: We changed it to the “good knowledge” (line 38)

• “line 39: 98% were ethical conscious in their practices – it is better to state 98% had good ethical practices”

Response: We corrected the sentence in (line 39)

• “line 44: nurses’ practice with high ethical standards – needs to be rephrased”

Response: We rephrased the sentence as appropriate (lines 44&45)

• “lines 78-80: What is the reference /basis for these statements?”

Response: We provided the references to these statements

• “lines 95-96: rephrase statement”

Response: We corrected the statement

• “lines 90-115: Sentences look repetitive. They may be condensed. syntax errors to be corrected. it seems that problems/concerns with nurse patient communication have been interchangeably used with ethical behaviour. This is not appropriate & needs to be corrected.”

Response: we restructured these sentences

• “line 119: How was sample size calculated?”

Response: We used all the nurses in the three facilities, which we described under the method section.

• “line 130: of the 42 items – only details of 30 items have been mentioned in section A & B”

Response: we actually used 32 items, which are accounted for in the descriptions

• “lines 164-168: grammatical corrections needed”

Response: we provided the corrections as needed

• “lines 190-192: how were knowledge, attitude & practice scores aggregated as continuous scores? why were the variables not analysed as categorical variables?”

Response: These explanations are provided under the data analysis (lines 168-185)

• “line 193: what is a latent variable?”

Response: We did provide a clear description for this.

• “line 194: what is mixture of variables? – it is preferable to utilize appropriate statistical terms.”

Response: We provided an appropriate description for that.

• “line 195: p value<0.05”

Response: We made the change as required.

• “what software was used for data analysis?”

Response: The correction has been made, as we used SPSS software, version 20.0.

• “line 200: high knowledge – to be corrected”

Response: We change it to good knowledge

• “line 208-209: table title needs to be reworded. level of ethical knowledge is incorrect usage. tables can be reframed to depict key variables rather than reproducing the data output tables”

Response: We change the table tittle as suggested

• “line 225: CHN/RCN/RMN – the explanation for the abbreviated terms need to be provided as footnotes under the table”

Response: We provided the full meaning of the abbreviations

• “lines 230-232: it is preferable to classify knowledge as good & poor”

Response: We change the phrases good and poor knowledge

• “line 311: high level of ethical knowledge to be replaced by good knowledge about ethics”

Response: We changed the sentence to reflect “goof knowledge”

• “line 325: who could not be a presentation of the all nurses in Ghana. – needs to be rephrased as not representative of all nurses in Ghana”

Response: This sentence has been corrected appropriately

• “lines 331-332: sentences to be rephrased”

Response: Sentences have been corrected

• “lines 334-337: please substantiate your claim on attitudes & patient recovery”

Response: We provide references and examples in support of the claims

• “lines 357-358: syntax error to be corrected”

Response: The correction was made

• “lines 368-370: sentences need to be rephrased”

Response: Sentences have been corrected

• “line 379: conclusion- should summarize key findings of the study in 3-4 sentences”

Response: We summarized the key findings appropriately

• “lines 380-383: may be removed from conclusions”

Response: We removed the sentences as suggested.

• “lines 384-388 avoid usage of term – high ethical standards”

Response: We change to good knowledge

• “lines 395-396: sentences need to be rephrased?”

Response: Sentences have been corrected

• “lines 391-393: are limitations of the study & not conclusions”

Response: These sentences have been removed

• “line 414: syntax error”

Response: We did correct this sentence
