# Supplementary material for: Ethics in healthcare: Knowledge, attitude and practices of nurses in the Cape Coast Metropolis of Ghana
Source: PLoS One. 2022 Feb 16;17(2):e0263557. doi: 10.1371/journal.pone.0263557 (PMC8849504; doi:10.1371/journal.pone.0263557)
Supplement: Attachment — Submitted filename: Response to Reviewers Comments.docx [file pone.0263557.s004.docx]

**Respond to Comments from the Reviewer**

We are very grateful to reviewers for the thorough review done so far on our paper. We provide the following responses to the comments. We indicated our corrections in Red Link in the main document (Revised Manuscript with Track Changes).

1. Updated references

Response:

2. Line 44: Good high ethical standards is not appropriate phrase. Please correct the statement.

Response: We corrected this, by deleting “high”.

3. Line 103: Improperly carried out ethical practices in their routine care like insulting patients. Please rephrase.

Response: we corrected this sentence, lines 102-103

4. Lines 125-126: It is preferable to state that all the 326 nurses working in the municipality hospitals were included in the study.

Response: This sentence is changed accordingly, line 124

5. Tables 2, 3, 4: Titles can be modified to improve understanding.

Response: Titles have been modified accordingly, lines 258-259, 276-277, 292-293

6. Line 330: The finding again indicates a high positive attitude- avoid usage of phrases like high positive attitude.

Response: We introduced “good” attitude in the sentence, line 324

7. Line 333: Oceanian – spell check for error.

Response: We correct, “Oceania”, line 327

8. Lines 369-372: Sentences are grammatically incorrect. Kindly rephrase.

Response: We corrected the sentences for understanding, lines 362-364
